# Phthalate exposures in a Canadian birth cohort at three months of age: the CHILD study

**DOI:** 10.1186/1710-1492-10-S1-A56

**Published:** 2014-03-03

**Authors:** Huan Shu, Ryan Allen, Carl-Gustaf Bornehag, Michael Brauer, Jeff Brook, Bruce Lanphear, Sheela Sathyanarayana, Malcolm Sears, Leilei Zeng, Tim Takaro

**Affiliations:** 1 Department of Health Sciences, Karlstad University, Karlstad, Sweden; 2 Faculty of Health Sciences, Simon Fraser University, Burnaby, Canada; 3 School of Population and Public Health, University of British Columbia, Vancouver, Canada; 4 Environment Canada, Canada; 5 Department of Pediatrics, University of Washington, Seattle, USA; 6 Department of Medicine, McMaster University, Hamilton, Canada; 7 Department of Statistics and Actuarial Science, University of Waterloo, Waterloo, Canada

## Background

Exposure to phthalates has been associated with the development of wheeze and asthma. While infants may be exposed via multiple routes, the sources of infant exposure aren’t fully understood.

## Methods

We employed the Canadian Healthy Infant Longitudinal Development (CHILD) Study, a multicentre, longitudinal, population-based birth cohort with 3,300 children to identify sources of phthalate exposures in infants. For the first 1,539 CHILD participants we examined associations between 6 urinary phthalate metabolites, measured at 3 months of age and corrected for specific gravity, with 90 variables characterizing the indoor environment, including furnishings, household care products and personal care products. Univariate, Bivariate, and Tobit regression were used for modeling. We also examined the relationship of urinary phthalates with socio-demographic characteristics and breastfeeding.

## Results

Overall, there were 32 variables associated with higher concentrations and 20 inverse associations. We found higher urinary phthalates among children whose families used oven cleaners (Mono-n-butyl phthalate (β=7%, 95%CI: 2-15%), Mono-benzyl phthalate (β=15%, 5-26%), and Mono-ethyl phthalate (15%, 5-26%)) and air fresheners (Mono-n-butyl phthalate (4%, 2-10%), Mono-benzyl phthalate (10%, 5-15%), and Mono-ethyl phthalate (10%, 5-17%)), or who heated food in hard plastic (Mono-n-butyl phthalate (32%, 15-48%), Mono-2-ethyl-5-hydroxylhexyl phthalate (29%, 7-51%). Soft vinyl flooring was highly correlated with Mono-benzyl phthalate (58%, 35-91%). Mono-2-ethylhexyl phthalate, Mono-2-ethyl-5-hydroxylhexyl phthalate, and Mono-2-ethyl-5-oxohexyl phthalate concentrations were lower in children who were breastfed. Household income was inversely associated with Mono-2-ethyl-5-oxohexyl phthalate concentrations.

## Conclusions

Our analysis demonstrated higher levels of phthalate metabolites associated with use of household product and plastics. The identification of these exposures as possible contributors to phthalate body burden in three-month-old children is an important step in exposure categorization and supports efforts to reduce exposure.

